# Primary Sjögren’s Syndrome in an Adolescent With Bilateral Multiple Parotid Glands Calcifications and Cysts: A Case Report

**DOI:** 10.7759/cureus.70509

**Published:** 2024-09-30

**Authors:** Saba' A Jarrar, Shawkat Altamimi, Reem Hasweh

**Affiliations:** 1 Otolaryngology, Al-Balqa Applied University, Al-Salt, JOR; 2 Radiology, Al-Balqa Applied University, Al-Salt, JOR

**Keywords:** acute parotitis, parotid calcification, parotid swelling, recurrent parotitis, sjogren’ s syndrome

## Abstract

Sjögren's syndrome (SS) is an autoimmune disorder that usually affects middle-aged females. Juvenile SS is of unknown prevalence. The classic presenting symptoms in adults are mouth and eye dryness, while in children and adolescents, recurrent parotitis is more often the presenting symptom with the absence of sicca symptoms. We present the case of a 17-year-old girl with recurrent bilateral parotid swelling for the last eight months. The parotid CT scan showed prominent parotid glands associated with multiple tiny calcifications and cyst formation. Serologic studies showed significantly elevated Ro/SSA antibodies. A lip biopsy showed periductal lymphocytic infiltration. As the patient fulfilled the diagnostic criteria for SS, she was subsequently referred to a rheumatologist for specialized management. This report aims to focus on the variation in the presentation of SS in younger age groups among different specialties that deal with these cases initially and to diagnose SS as early as possible to improve the outcome and reduce complications.

## Introduction

Sjögren's syndrome (SS) is a chronic systemic autoimmune disease characterized by autoimmune-induced inflammation of exocrine glands, particularly the lacrimal and salivary glands, leading to dry eyes and mouth. The clinical manifestations of SS include both exocrine gland involvement and extraglandular disease manifestations. There are two forms of SS: primary SS, which is not associated with other diseases, and secondary SS, which overlaps other autoimmune conditions, most commonly rheumatoid arthritis (RA) and systemic lupus erythematosus (SLE) [1]. In both primary and secondary SS, dry eye (keratoconjunctivitis sicca) and dry mouth (xerostomia) are caused by decreased exocrine gland function. Other disease manifestations can affect other organs such as skin, joints, liver, kidneys, lungs, heart, GI tract, gynecologic, and nervous systems [2].

This syndrome typically affects middle-aged women. The prevalence of primary SS is approximately 0.05% to 0.23% in the general population. It can also affect children and adolescents, but the true prevalence in children and adolescents is not known [3]. The clinical presentation differs significantly in children and adolescents as compared to adults, which makes the diagnosis of SS in pediatrics and adolescents challenging [4]. Eye and mouth dryness are less common in childhood, especially at the onset of the disease. Recurrent parotitis is frequently the first and most predominant symptom [4].

## Case presentation

A 17-year-old girl presented with recurrent bilateral facial swelling and discomfort, with more prominence on the right side for the last eight months. Her facial swelling worsened with eating and subsided gradually afterward. The episodes of facial swelling were not associated with fever or dental problems. She denied any malaise, night sweats, weight loss, skin rash, oral or ocular dryness, or any joint pain. Antibiotics and analgesics were used to manage these episodes, but symptoms used to recur shortly afterward. The patient's past medical and surgical history was uneventful. Her mother has hypothyroidism, but it was not confirmed to be autoimmune in origin.

**Figure 1:**
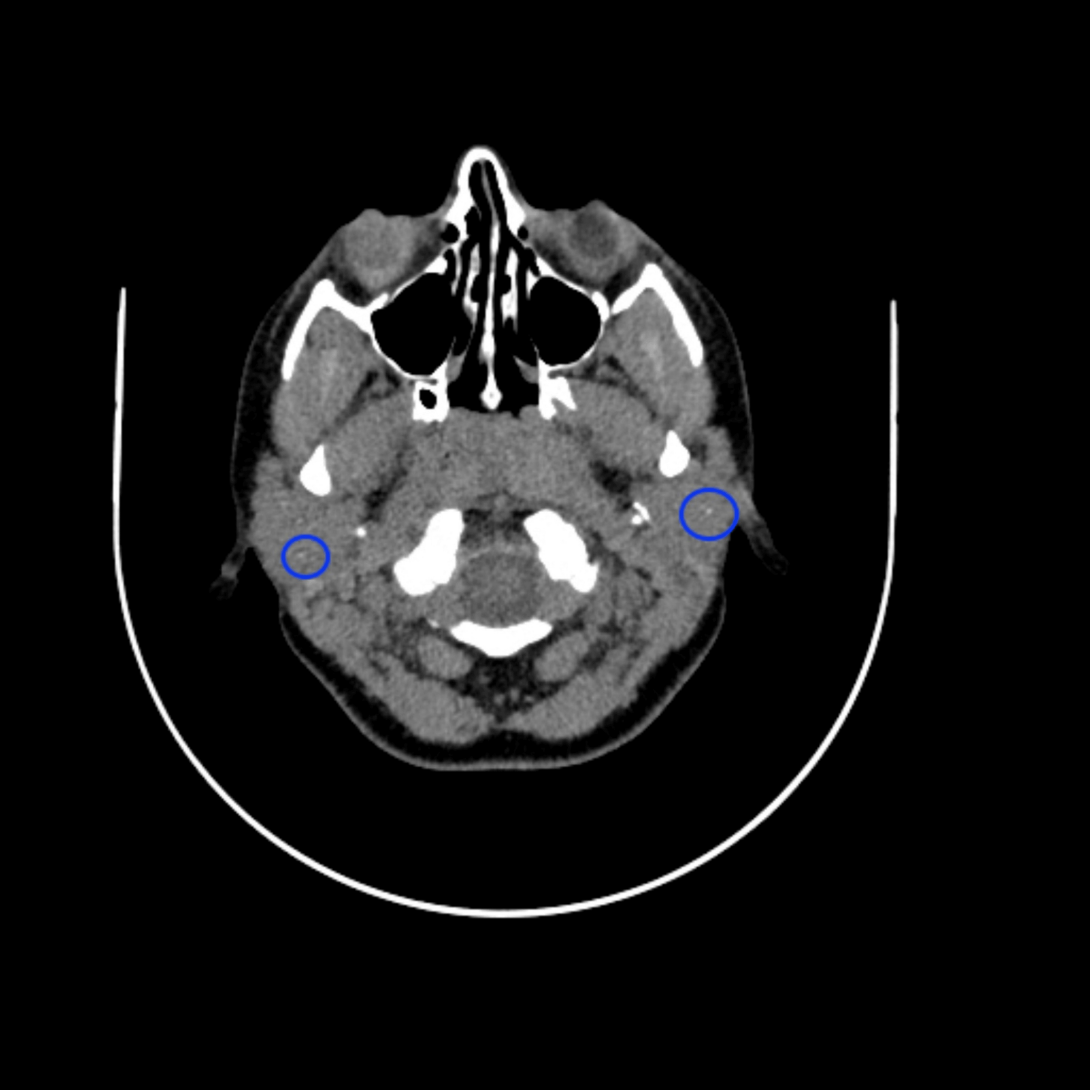
Precontrast CT shows tiny foci of calcifications within the parotid gland bilaterally. Foci of calcifications are encircled by blue rings. Precontrast CT shows tiny foci of calcifications within the parotid gland bilaterally. Foci of calcifications are encircled by blue rings.

**Figure 2:**
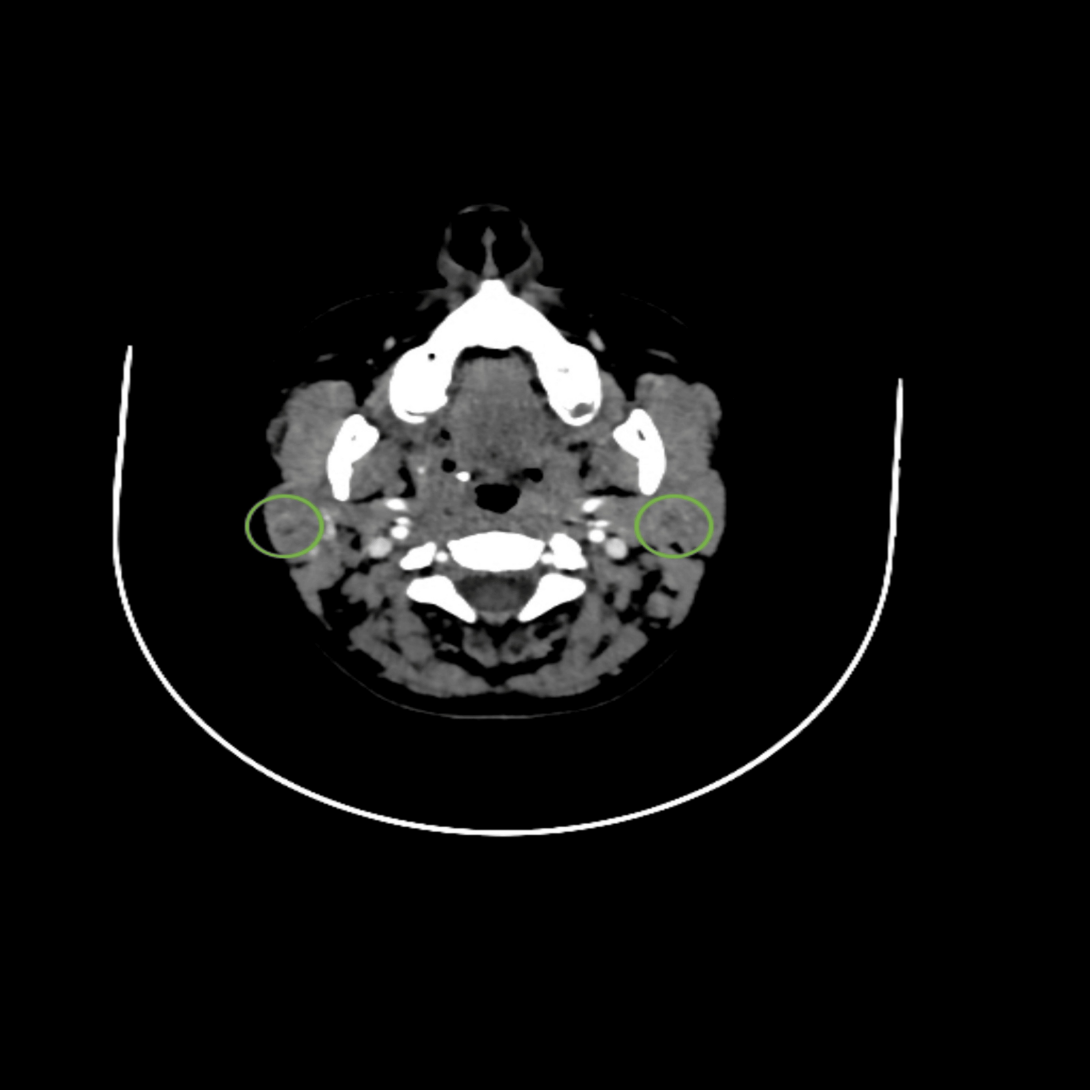
Postcontrast CT showing cystic changes in parotid glands bilaterally. The cystic changes are highlighted by the green circles.

Physical examination showed a right parotid lump measuring 1 cm x 1 cm; the lump was smooth and firm with no redness, tenderness, hotness, or fluctuation. Her oral exam showed wet mucous membranes and tongue, no signs of decreased salivary flow, and no signs of tooth decay or oral thrush. Saliva was coming out from the opening of the parotid duct on parotid massage. There were no palpable cervical lymph nodes. The rest of her ear, nose, and throat exam was unremarkable. A CT scan with contrast showed prominent parotid glands associated with multiple tiny calcifications predominantly on the right side (Figure 1) associated with tiny hypodensities representing mostly tiny cysts (Figure 2).

Based on the findings from the CT scan, SS was suspected, and a series of blood tests were subsequently conducted for further evaluation. The patient's hemoglobin was 12.1 g/dL, and WBC count was 5.77 x10^9/L. She had elevated ESR 40 mm/hr, a positive rheumatoid factor 32 mg/L, elevated anti-nuclear antibodies (ANA) of homogenous pattern 1/80, and a significantly elevated anti-Ro/SS-A >100 kU/l (Table 1). She had negative anti-double-stranded DNA and anti-La/SS-B.

**Table 1:** Blood tests

Test	Result	Normal range
ESR	40 mm/hr	0-20 mm/hr
Rheumatoid factor	32 mg/L	Negative < 8 mg/L
Anti-nuclear antibodies (ANA)	1/80 homogenous pattern	Negative < 1:40
Anti Ro/SS-A antibody	>100 kU/l	< 0.30 kU/l

Sjögren's syndrome was suspected based on the clinical findings, blood test results, and CT findings. The patient's case was reviewed in consultation with a rheumatologist, who recommended a lip biopsy to confirm the diagnosis. Consequently, a minor salivary gland biopsy (lip biopsy) was performed, revealing multiple foci of periductal lymphocytic infiltration. Based on these findings, the diagnosis of SS was confirmed. She was referred to a rheumatologist who started her on oral prednisolone (10 mg/day), hydroxychloroquine (200 mg/day), and methotrexate (10 mg/week) based on her symptoms and the frequency and severity of her attacks. The dose of methotrexate will be adjusted according to the patient's response and symptoms. She is being followed up by her rheumatologist. Her attacks of parotid swelling have become less painful and less frequent within the first three months of treatment. Further regular follow-up is arranged to monitor her response and adjust her medications accordingly.

## Discussion

Sjögren's syndrome is a chronic autoimmune disease mainly involving the exocrine glands. It commonly affects middle-aged women. The most common presenting symptoms are keratoconjunctivitis sicca and xerostomia. The incidence of juvenile SS is rare but is becoming more evident. Only 5% of adults with SS reported that the onset of their symptoms was earlier than the age of 12. Juvenile SS affects females more than males with a ratio of 7:1 and the mean age of onset is 10 years. Juvenile SS presents initially with recurrent parotid swelling rather than sicca symptoms [5]. The information about juvenile SS is still limited; therefore, standardized diagnostic criteria are not available, making the diagnosis of juvenile SS challenging. Common autoantibody markers like anti-Ro/SS-A and anti-La/SS-B that are used in adult SS diagnosis are negative in about 26% of juvenile SS, making them of limited value in pediatric patients [5].

Primary SS in adults can be diagnosed according to the Revised International Classification Criteria for SS from the American-European Consensus Group if four of the following six criteria are met: presence of ocular symptoms, presence of oral symptoms, evidence of keratoconjunctivitis sicca, focal sialadenitis upon minor salivary gland biopsy, instrumental evidence of salivary gland involvement, and the presence of SSA or SSB antibodies. These criteria are not accurate when applied to juvenile SS due to the variation in the presenting symptoms in children and adolescents. Studies showed that up to 75% of juvenile SS patients do not present with eye and mouth symptoms but have similar pathological and laboratory findings as adult cases [5].

Juvenile SS is uncommon in children but should be considered in cases of recurrent parotid swelling or recurrent parotitis. The mean age of presentation of juvenile SS is seven to 14 years; it has a marked predilection for females. The most common systemic manifestation of SS in children is leukopenia. Other systemic manifestations in adults can also be seen in children, including purpura, interstitial lung disease, arthritis, arthralgia, renal tubular acidosis, gastrointestinal disease, splenomegaly, and frequent upper airway infections. The most frequent serologic studies findings include positive ANA, which has been reported in more than 80% of patients in most series of childhood SS; anti-Ro antibodies (SSA) that are found in more than 70% of patients in most reports; or anti-La (SSB) antibodies, which are detected in 30% to 70% of cases; and elevated ESR. Rheumatoid factor presence and hypergammaglobulinemia in SS are seen rarely in children compared to adults. Other hematological findings in children include anemia, leukopenia, and thrombocytopenia [6,7].

Pathological changes in the salivary glands can be evaluated using various imaging modalities such as ultrasonography, CT, MRI, and magnetic resonance sialography. On MRI, changes like multiple, mixed, hypointense, and hyperintense foci 'salt and pepper appearance' can be seen and are suggestive of SS [8]. Salivary gland CT is accurate and reliable in the diagnosis of SS. Parotid CT findings that are specific for SS are heterogeneity, abnormal diffuse fat tissue deposition, and diffuse punctate calcification [9]. A small proportion (5.8%) of patients had presented with well-circumscribed cystic lesions, confirmed as lymphoepithelial cysts [8]. A minor salivary gland biopsy is one of the required criteria for diagnosis of SS, especially in the absence of eye and mouth dryness symptoms. The characteristic appearance of at least one focus of inflammation of greater than 50 cells is consistent with SS diagnosis [7].

The management of SS should be under a multidisciplinary team that includes a rheumatologist, an otolaryngologist, an ophthalmologist, and a dentist. Regarding the parotid involvement in SS, the management is symptomatic; the calcifications are usually tiny and within the parenchyma of the gland. Management usually involves sialagogues, secretagogues such as pilocarpine, and short-term topical corticosteroids to relieve sicca symptoms if present. If parotid stones fail to be managed conservatively, surgical interventions are indicated. Surgical interventions include stone removal under sialendoscopy and shock-wave lithotripsy under ultrasound guidance. Parotidectomy should be the last resort and only in patients with refractory symptoms or failed minimally invasive techniques [10].

## Conclusions

The diagnosis of juvenile SS is challenging due to its rarity and the different presenting symptoms in children and adolescents, which mainly involve the parotid gland, such as recurrent parotid swelling or recurrent parotitis, rather than the sicca symptoms that are more common as presenting symptoms in adults. Sjögren's syndrome should be considered in the differential diagnosis of recurrent parotid swelling in children and adolescents even in the absence of sicca symptoms. Awareness of this variation in the presentation of SS in the younger age groups should be spread among otolaryngologists, ophthalmologists, maxillofacial surgeons, and dentists because these doctors are often the initial providers for patients with SS. Early diagnosis and management provided by an interdisciplinary team consisting of rheumatologists, otolaryngologists, ophthalmologists, and dentists will help improve the course of the disease and reduce complications.
